# Heavy eye syndrome in a myopic patient with prior scleral buckle surgery for rhegmatogenous retinal detachment: Differential diagnosis and management

**DOI:** 10.1016/j.ajoc.2022.101418

**Published:** 2022-02-15

**Authors:** Motazz Alarfaj, Abdullah Al-Owaid, Hamdah Alkhaldi, Sahar Elkhamary, Gorka Sesma

**Affiliations:** a Pediatric Ophthalmology and Strabismus Division, King Khaled Eye Specialist Hospital, Riyadh, Saudi Arabia; b Department of Ophthalmology, Imam Abdulrahman Bin Faisal University, Dammam, Saudi Arabia; c Diagnostic Imaging Department, King Khaled Eye Specialist Hospital, Riyadh, Saudi Arabia

**Keywords:** Heavy eye syndrome, Loop myopexy, Scleral buckle

## Abstract

**Purpose:**

To present a challenging case of heavy eye syndrome (HES) in a 56-year-old female who previously underwent scleral buckle surgery in both eyes.

**Observations:**

Ophthalmic tests indicated a diagnosis of HES, confirmed using pre and postoperative magnetic resonance imaging (MRI). A silicone band loop myopexy was performed, successfully improving large angle esotropia at primary position and motility.

**Conclusions and Importance:**

MRI is essential to correctly identify HES, allowing a tailored surgical intervention that may lead to better outcomes for patients. Up to our knowledge, this is the first reported case of scleral fixated silicone band loop myopexy for HES in a previously scleral buckled patient.

## 1 Introduction

Heavy eye syndrome (HES), or myopic strabismus fixus, is an acquired progressive type of esotropia associated with severe myopia. Patients with HES present with eyes that are in esotropia with limited abduction due to elongation of the axial length and herniation of the sclera between the superior rectus (SR) and lateral rectus (LR).^1^ Inferior displacement of the LR causes hypotropia and limits abduction, while nasal displacement of the SR causes esodeviation and restricts supraduction. Loop myopexy is used to modify the SR and LR positions, thus restoring their physiological function.^2^, ^3^, ^4^, ^5^

## 2 Case report

**Fig. 1:**
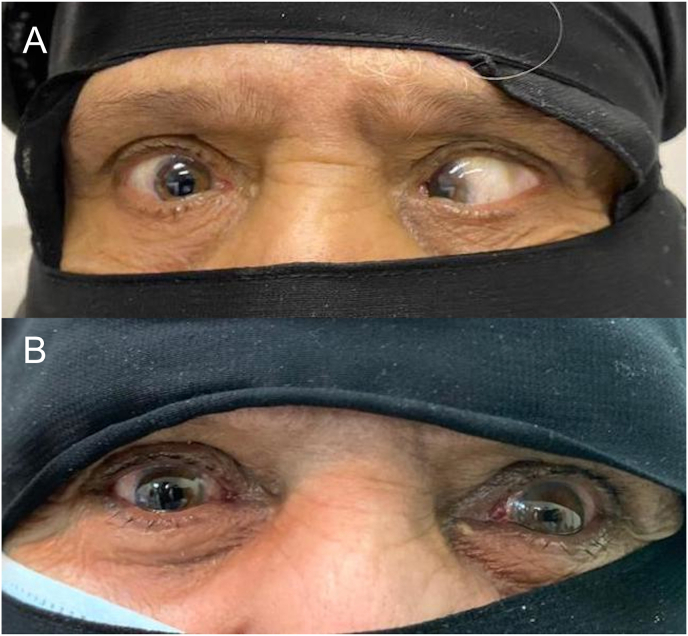
(A) Preoperative photograph showing a large angle esotropia. (B) 4-weeks postoperative photo showing mild exotropia at primary gaze.

**Fig. 2:**
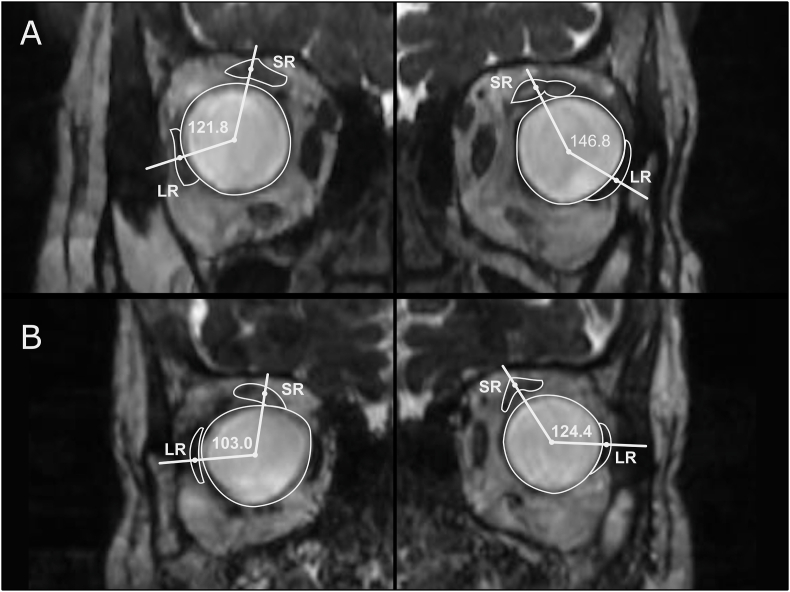
(A) Preoperative high-resolution FIESTA study coronal cut image of the orbit showing superotemporal globe herniation with redundant intermuscular septi, tight apposition of the lateral rectus (LR) to the globe, and subsequent displacement of the superior rectus (SR) nasally and the (LR) inferiorly. The coronal images of the right and left globes were taken at different levels to precisely locate the centroids of the muscles in relation to each globe. The angle is measured between the centroids of the SR and the LR to the globe. (B) Postoperative images showing the angles have decreased by about 18.8° in the right eye, and 22.4° in the left eye.

**Fig. 3:**
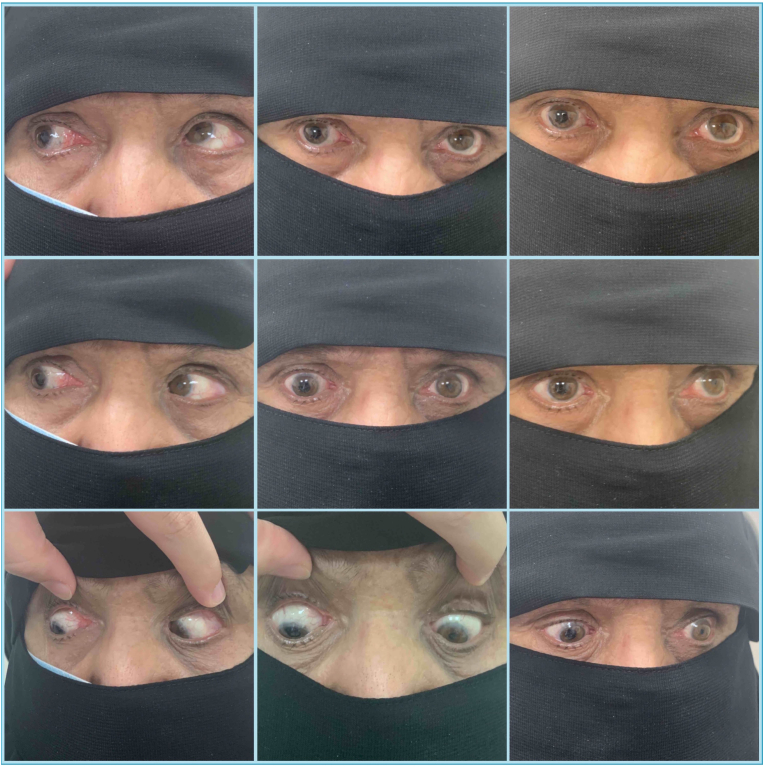
6-Months composite postoperative photo showing all nine gazes with small exotropia at primary gaze, mild adduction limitation of the right eye, and marked improvement in her abductions.

A 56-year-old female presented to our strabismus clinic complaining of progressive esotropia over several years (Fig. 1). She had a history of retinal detachment in both eyes, which had been treated with a scleral buckle more than 15 years prior to presentation. However, the esotropia was present about 5 years before the retinal procedures. Upon examination, her visual acuity was 20/100 in the right eye and 20/300 in the left eye. Anterior segment examination showed long standing mid dilated pupil related to previous ocular procedures in the right eye with aphakia and a normal left eye with early cataractous changes. Fundus examination showed degenerative myopic changes and a scleral buckle of excellent height. Ocular motility examination showed limitation of elevation −2 in both eyes. The right eye had moderate abduction limitation −2, while the left eye had severe abduction limitation −3. In primary gaze, she had an angle esotropia of >90Δ and a small left hypotropia 6Δ (Fig. 1). The axial length was measured 29.8 mm in the right eye and 30.5 mm in the left eye. Magnetic resonance imaging (MRI) confirmed the diagnosis (Fig. 2A). A high-resolution T2-weighted MRI.3T scanner (GE Discovery 750W MRI; General Electric, Milwaukee, WI, USA) was used. The MRI data was transferred to the picture archiving and communication system (PACS) in the original digital imaging and communications in medicine (DICOM) format through AGFA workstation. Orbital coronal MRI images were evaluated. The LR and SR muscles positions were determined relative to the globe center from quasicoronal images and correlated with LR-SR band structure. The LR-SR dislocation angle was defined as the angle formed between the centroid of LR and SR and the centroid of the globe, in accordance with the method of Yokoyama et al.^2^ The LR-SR band was qualitatively assessed as thinning, discontinuity, or displacement by examining adjacent quasicoronal image planes. The treating physician decided to plan for loop myopexy with a scleral band in both eyes plus bilateral medial rectus (MR) recession. Under general anesthesia, forced duction test showed bilateral tightness in abduction and supraduction. Intraoperatively, there were encircling style 240 scleral buckles in both eyes placed almost 15 mm from the limbus. The MR was approached through a fornix incision and recessed 8 mm using a hang-back technique. Then, through a superotemporal fornix incision, the nasally displaced SR was hooked and isolated from the surrounding attachments. The same was done for the LR, which had been displaced inferiorly. Manipulation of the existing scleral buckle was avoided to prevent avoidable retinal complications. Afterward, a scleral tunnel was made 12 mm from the limbus in the superotemporal quadrant, posterior to the existing scleral buckle. A style 240 silicone band was passed under the SR and then through the tunnel and under the LR. The two ends of the silicone band were tightened and pulled in opposite directions with a silicone sleeve style 70 to approximate both muscles. The ends of the silicone band were trimmed, and the conjunctiva was closed using 8-0 Vicryl sutures. This exact procedure was repeated for the other eye. At 6 months follow-up, she had 8Δ exotropia, and good convergence with marked improvement in her abduction (Fig. 3). MRI was repeated and showed the angles measured between the centroids of the SR and the LR to the globe have decreased by about 18.8° in the right eye, and 22.4° in the left eye (Fig. 2B).

## 3 Discussion

HES is a consequence of abnormal muscle positioning, rather than abnormal muscle force. Correctly diagnosing HES is essential to identify the correct type of procedure to use to avoid unfavorable outcomes. For example, resection of an inferiorly displaced LR worsens the deviation and creates a stronger depressing force, making classical recession and resection a poor surgical choice.^1^ High myopia is a well-known risk factor for retinal detachment, which is often treated using scleral buckle surgery. Strabismus, diplopia, and ocular motility disorders are known side effects following scleral buckle surgery, which have been attributed to scarring, extraocular muscles disinsertion, or slippage.^6^, ^7^, ^8^ However, our patient had a high angle of esotropia that was present long before the scleral buckle surgery.

All loop myopexy procedures described in the literature are based on the same concept and share a common goal: to restore the displaced muscles to their normal physiological locations. Various surgical techniques and modifications have been reported, all of which were based on three main procedures: first, Yokoyama's procedure, which is a union between the SR and the LR pullies using nonabsorbable 5–0 polyester sutures with MR recession if contracture is present^2^; second, Yamada's procedure, which combines hemitransposition of the temporal SR and the superior LR to the sclera combined with MR recession^3^; and third, the partial Jensen's procedure, in which the SR and LR are split in half from their insertion to past the equator and the approximal halves are joined together with a 5–0 Dacron suture.^4^ Most cases in literature detail minor alterations to the surgical techniques mentioned above, such as additional MR recession, use of botox, scleral fixation, type of suture used, or the use of silicone bands.^5^ Scleral fixation provides better stability at the expense of increased risk of perforation, whereas silicone band without scleral fixation has the risk of migration.^9^

MRI provides valuable information in cases that present with strabismus after a scleral buckle procedure and can differentiate between multiple mechanisms that lead to strabismus, allowing for better surgical planning and outcome.^8^ MRI can also help to distinguish HES from sagging eye syndrome (SES). Esotropia in SES can occur in both myopic and nonmyopic eyes and is due to degeneration of LR-SR band with subsequent inferior displacement of the LR. In HES, LR-SR degeneration is secondary to the superotemporal prolapsing globe. The LR in HES is tightly apposed to the globe. MRI in HES is associated with a superotemporal globe shift and the mean angle between the SR and LR in is 121° ± 7° whereas in SES is 104° ± 11°.^10^ The larger angle measurements found in HES is supported by Yokoyama et al. who reported the angle between the SR and LR in HES is 180° ± 31°.^2^ The angle measurements in our patient were 121.8° in the right eye and 146.8° in the left eye. Also, SES is associated with degenerative aging changes like blepharoptosis and deepening of the sulcus, which our patient did not demonstrate.

Most strabismus occurring after retinal detachment surgeries are transit and resolve within 3–6 months with only 5%–25% of the cases are persistent. Different etiologies have been described that include sensory deviation secondary to poor vision, direct muscle injury, redirection of muscle force vectors, and scarring of Tenon's capsule and adhesions formation.^11^ Our patient had a history of large angle esotropia about 5 years before the scleral buckle surgery. This ruled out the differential diagnosis of acquired esotropia as a complication from the scleral buckle. Identifying the cause of the strabismus helps to avoid premature removal of the scleral buckle that supports the retina. If MRI shows superotemporal globe shift, large angle of displacement between the SR and LR muscles' paths, and tightly apposed LR to the globe, HES is confirmed, and the surgical methods described here are the treatments of choice. There are several challenges in the management of HES with prior history of scleral buckle surgery. The presence of a previous buckle surgery makes it difficult to perform a loop myopexy due to the scarring process, adhesions, and anatomical changes. In addition to being the surgeon's preference, fornix incision was selected to provide better access posteriorly and to avoid extra dissections associated with the limbal incisions. This improves cosmesis and decreases difficulty of reoperations if needed in the future. Fornix incision helped avoid anterior manipulation of the conjunctiva and Tenon's capsule to prevent anterior migration of the existing scleral buckle and the other rare, but serious, complications such as exposure and extrusion. Up to our knowledge, this is the first reported case of scleral fixated silicone band loop myopexy for HES in a previously scleral buckled patient.

## Patient consent

Written consent was obtained from the patient.

## Funding

No funding or grant support.

## Authorship

All authors attest that they meet the current ICMJE criteria for Authorship.

## Conflicts of interest

No conflict of interest exists.

## Funding

No funding was received for this work.

## Intellectual property

We confirm that we have given due consideration to the protection of intellectual property associated with this work and that there are no impediments to publication, including the timing of publication, with respect to intellectual property. In so doing we confirm that we have followed the regulations of our institutions concerning intellectual property.

## Research ethics

We further confirm that any aspect of the work covered in this manuscript that has involved human patients has been conducted with the ethical approval of all relevant bodies and that such approvals are acknowledged within the manuscript.

IRB approval was obtained (required for studies and series of 3 or more cases).

Written consent to publish potentially identifying information, such as details or the case and photographs, was obtained from the patient(s) or their legal guardian(s).

## Authorship

All listed authors meet the ICMJE criteria.  We attest that all authors contributed significantly to the creation of this manuscript, each having fulfilled criteria as established by the ICMJE.

We confirm that the manuscript has been read and approved by all named authors.

We confirm that the order of authors listed in the manuscript has been approved by all named authors.

We confirm that the order of authors listed in the manuscript has been approved by all named authors.

## Declaration of competing interest

The following authors have no finical disclosures. (MA, AA, HA, SA, GS).
